# The Visit of Metchnikoff

**Published:** 1906-06-02

**Authors:** 


### The Visit of Metchnikoff.

Public attention has been drawn to the visit of a great scientist to England. The word scientist, however, has not perhaps much attraction for the great majority even of highly educated people. We once heard a learned and greatly respected judge, in the course of an interesting address, remark that the writings of most scientists were like "a collection of facts hurled at the reader." That there are such writings by men engaged in scientific investigation no doubt may be true, but we should not feel inclined to allow that any man who can do no more than throw his facts in a shower upon the world, however weighty they in themselves may be, will ever earn the title of great. Power of observation is a valuable quality, but power of observation alone may add to mystery rather than give illumination. It is possible for a man to be swamped by his facts. A scientific man, or any man of learning, noteworthy only on account of the possession of exceptional knowledge may be looked upon with some feeling of awe by others less erudite, but he will never be a source of inspiration to his fellows. The man of power and influence is he who can, so to speak, soar above his facts, take a bird's eye view of them, and piece them if possible into one harmonious whole. Such a bird's eye view will probably show him what is lacking and where there is need for renewed observation and careful investigation. To examine thus, to see in connection with facts not only the present but the future, requires the power of imagination, and the placing of his observations in their true relation to one another necessitates reasoning faculties of a high order. The great scientist brings to bear upon his work not only keen observation, but a brilliant imagination, and the most acute powers of reasoning.

In Professor Metchnikoff we meet with one of these masterly minds which have the power to bring through intricate paths the unknown into the light of day. Metchnikoff has added to the knowledge of medicine in many fields, but possibly his work on the nature of immunity has had the most far-reaching influence. That the human being possesses the power of recovery from illness is well known, and that it possesses the power, in the case of some diseases, after recovery has taken place of resisting further attacks of the same disease, is also a matter of common knowledge, but the wonderful processes at work in accomplishing this end have until recently remained hidden from the eyes of all observers. Metchnikoff may be said to have taken the first step towards explaining the mystery when he described the power of the white cells of the blood to take up and digest bacilli. This, however, proved to be only the first step. His work stimulated other investigators who also made valuable observations, some of which seemed to lessen the importance of the investigations of Metchnikoff; controversy was aroused, and controversy and investigation going hand in hand showed how almost incredibly complicated is apparently so simple a matter as the protection of our bodies against disease. It is true that the cells of the blood and other cells of the body play an important part in the destruction of invading bacilli, but the cells do not destroy micro-organisms only by taking them into their substance and digesting them. There are also not only the bacilli to be overcome and got rid of, but the poisons produced by these bacilli require to be neutralised. The digestion of bacilli and the neutralising of poisons may not appear to present questions for investigation of great intricacy, yet the study of the methods adopted by the body to accomplish these ends, has shown perhaps more than any other study of animal economy how astonishingly complicated are the many processes at work within us in health and disease. Exerting a profound influence upon, we may almost say presiding over and checking at every turn the investigations of numerous workers, one or two of them with intellectual gifts almost, if not quite, equal to his own, has been the genius of Metchnikoff. And this work it must not be imagined is of merely scientific interest. The body in many instances happily gets the better of the fight against disease, but we know only too well how often it succumbs in the conflict. The scientist first learns how the fight is carried on, his next aim is to discover the best means of supplying the body with the necessary weapons. The object he has in view is, however, not only to provide cure, but to establish prevention, that is to give power to ward off the first attack. Something in both of these directions has already been done, and the greater part of the basis upon which it is to be hoped the future superstructure will be built, has been laid by Metchnikoff.

